# The Controllable Mechanical Properties of Coiled Carbon Nanotubes with Stone–Wales and Vacancy Defects

**DOI:** 10.3390/nano13192656

**Published:** 2023-09-27

**Authors:** Zhiwu Bie, Yajie Deng, Xuefeng Liu, Jiaqi Zhu, Jixiao Tao, Xian Shi, Xiaoqiao He

**Affiliations:** 1 Department of Architecture and Civil Engineering, City University of Hong Kong, Tat Chee Avenue, Hong Kong; zwbie2-c@my.cityu.edu.hk (Z.B.); jqzhu3-c@my.cityu.edu.hk (J.Z.); bcxqhe@cityu.edu.hk (X.H.); 2 School of Aerospace Engineering and Applied Mechanics, Tongji University, Shanghai 200092, China; 3 Department of Mechanics and Aerospace Engineering, Southern University of Science and Technology, Shenzhen 518055, China; xfliu6-c@my.cityu.edu.hk (X.L.); jixiaotao2-c@my.cityu.edu.hk (J.T.); 4 School of Civil Engineering, Suzhou University of Science and Technology, Suzhou 215009, China; shixian@usts.edu.cn; 5 Center for Advanced Structural Materials, City University of Hong Kong Shenzhen Research Institute, Shenzhen 518057, China

**Keywords:** defected coiled carbon nanotubes, Stone–Wales defects, vacancy defects, molecular dynamics

## Abstract

Coiled carbon nanotubes (CCNTs) as a promising nanometer scale spring are investigated for the effect of the defects on the tensile mechanical properties of CCNTs by using molecular dynamics (MD) simulations. Six samples of defective CCNTs are constructed by introducing the defects in the different positions. The results show an obvious decrease in the spring constant and elastic limit of defective CCNTs, which results in the lower energy storage ability during the elastic range compared with the perfect CCNTs. However, the defected CCNTs exhibit better ductility (138.9%) and higher energy absorbing ability (1539.93 J/g) during the fracture process since introduced defects change the deformation pattern. Furthermore, among the defected CCNTs, the stiffness (1.48~1.93 nN/nm), elastic limit (75.2~88.7%), ductility (108.5~138.9%), and deformation pattern can be adjusted by changing the position or the type of defects. This study firstly provides insight into the effects of Stone–Wales (SW) and vacancy defects on the mechanical properties of CCNTs, and the obtained results are meaningful for designing CCNTs with specified properties by introducing defects.

## 1. Introduction

As a three-dimensional carbon nanomaterial, CCNTs exhibit special mechanical, structural, and electrical properties that are suitable for application in nanometer-scale springs, sensors, resonators, actuators, energy storage devices, and mechanical devices to store or consume energy and reinforcement in composites [1,2,3,4,5,6,7,8,9].

CCNTs with different geometries have been fabricated in the experiments, and their properties have been explored extensively [1,10,11,12,13,14,15]. Hayashida et al. [5] found that CCNTs possess different spring constant (up to 0.6 N/m) and different Young’s modulus (up to 0.13 TPa) according to different geometrical properties, and Chen et al. [13] measured the maximum elastic strain (up to 33%) for CCNTs. Furthermore, through atomistic quantum methods, Liu et al. [16] obtained the spring constant from 15.37 to 44.36 nN/nm, and Young’s modulus is measured to be 3.43 to 5.4 GPa of CCNTs, which consists of CNT segment having the various chirality. Fonseca et al. [17] used the Kirchhoff rod model to successfully calculate Young’s modulus (up to 6.88 GPa) and Poisson’s ratio (0.27). By using the MD method, Wu et al. [18] investigated the effect of two types of defect (pentagon–heptagon defects and pentagon–octagon defects) and the number of strands on the mechanical properties of CCNTs, and the results showed that the toughness of CCNTs increased as the length of CNT segments decreased, and the spring constant increased with the increase of the number of strands. The stretchability, reversibility, and the effect of temperature on the mechanical properties of the tightly wound CCNTs were studied by Wu et al. [19]. The spring constant of 12.57 to 30.71 nN/nm was obtained in their works, and it was found that the maximum elastic strain decreased at higher temperatures. Then, the effect of the chirality of CCNTs was investigated by Wu et al. [20], and the results showed that the chirality did not affect the deformation pattern. Sharifian et al. [21] investigated the effect of geometrical properties on the tensile and compressive mechanical properties of CCNTs. Furthermore, Wu et al. [22] constructed entwined coiled carbon structures with single- to triple-helix, and the increase in the number of entwined helices caused the increase in stiffness. Bie et al. [23] investigated the tunable mechanical properties of CCNTs with different defect positions and geometries through three different construction procedures. Besides using MD methods, Ghaderi et al. [24] used the molecular structural mechanics method through finite element code to obtain the mechanical properties of CCNTs, and the results showed that both the spring constants and shear modulus increased along with the increase of the diameter of CNT segments. In addition, Ju et al. [25] studied the CCNTs constructed by double-walled CNTs and found that CCNTs with double-walled CNT segments exhibited better ductility than CCNTs with single-walled CNT segments.

All above-mentioned CCNTs can be seen as the perfect helical structures due to the fact that the defects in the CCNTs were only introduced into the joints of carbon nanotubes (CNTs) segments to generate the curves of helix [26,27,28]. However, there are defects (i.e., Stone–Wales and vacancy defects) observed experimentally in other parts of the carbon-based materials, including CNTs and graphene [29,30,31,32], which may be controlled by the irradiation during the growth [33]. The previous study showed that the mechanical properties of CNTs measured in the experiments are different from those obtained from theoretical studies [34,35]. This discrepancy was believed to be caused by the introduction of defects in the original carbon structures. The mechanical properties of carbon-based materials also depend on the position, the number, and the type of defects. For example, the stiffness, strength, and failure strain of the CNTs under tensile loads decreased obviously after introducing defects [36,37,38,39]. The decrease pattern was observed for the buckling forces under compressive loads [40]. Furthermore, the plastic deformation of CNTs under the tensile loads was believed to be caused by the slippage of the SW defects [37]. For CNTs with multiple defects, the mechanical properties decreased with the increase in the average number of defects [37,38,39]. It was also found that vacancy defects can reduce the properties of CNTs more significantly [37].

Since the structure of CCNTs is believed to be formed by introducing non-hexagonal defects in certain positions of straight CNT segments to generate the helix [1,26], the introduction of SW and vacancy defects in the CNT segments of CCNTs is believed to influence the mechanical properties obviously. However, up to date, all investigations of CCNTs only considered the essential defects that are used to generate the curves. The effect of SW defects and vacancy defects in the straight CNT segments on the mechanical properties of CCNTs has not been studied. In the present study, a perfect structure of CCNTs is constructed, and SW and vacancy defects are introduced into the different positions of CNT segments of perfect CCNTs. The effect of the types and positions of defects on the tensile mechanical properties of defected CCNTs are studied through MD simulations. Interestingly, unlike straight CNTs, where the defects can obviously reduce the mechanical properties, CCNTs with certain defects exhibit an increased pattern in the spring constant, energy absorbing capacity, and ductility. Furthermore, these mechanical parameters are found to be dependent on the type and position of defects, which is meaningful for understanding the mechanism of the influence of defects on the mechanical properties of CCNTs.

## 2. Molecular Models and Methods

**Figure 1:**
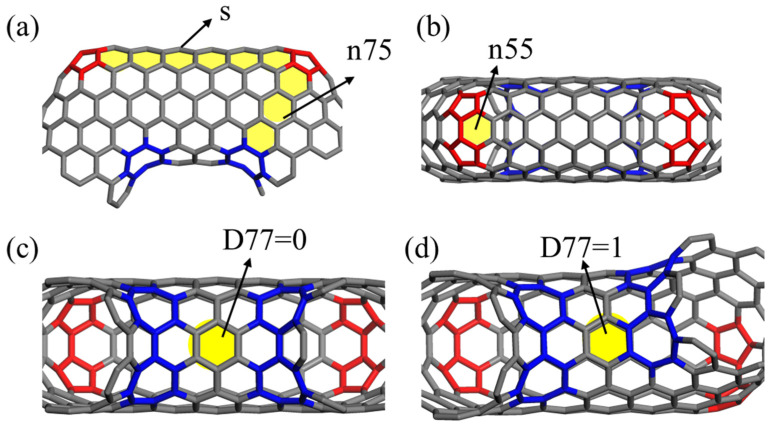
The diagram of indices (n75,n55,s,D77) of CCNT structure: (**a**) the indices of n75 and s, (**b**) the index of n55, (**c**,**d**) the index of D77.

According to the construction procedures in [16], the perfect CCNTs are constructed by introducing pairs of pentagon–heptagon defects into the straight CNTs with the chirality of (6,6) to generate the spiral structure. As shown in Figure 1, four indices are used to identify CCNTs, i.e., (n75,n55,s,D77), where n75 is the number of hexagons between heptagon and pentagon along the peripheral direction of CNT segments at the corner of helical CNTs, n55 is the number of hexagons between two pentagons, s is the number of hexagons between two pentagons along the axial direction of CNT segments, and the D77 is the number of hexagons between two heptagons of adjacent CNT segments, which is set to one. Hence, the CCNTs in this study are identified as (2,3,7,1). The geometrical properties of CCNTs can be adjusted by changing these four indices.

**Figure 2:**
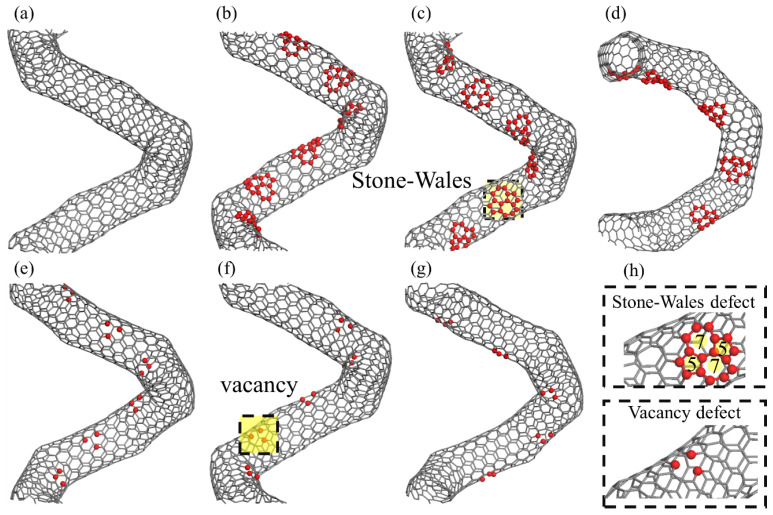
The atomic configurations for (**a**) perfect CCNTs, (**b**) (2,3,7,1)/sw1, (**c**) (2,3,7,1)/sw2, (**d**) (2,3,7,1)/sw3, (**e**) (2,3,7,1)/v1, (**f**) (2,3,7,1)/v2, and (**g**) (2,3,7,1)/v3. (**h**) is the illustration of the position of the introducing defects.

In addition to the essential pentagon–heptagon defects at the joints in CCNTs, the SW defects are constructed by rotating the sp2 bonds by 90° to form two pairs of heptagon–pentagon, and vacancy defects are introduced by deleting one atom in the CNT segments (Figure 2h). As shown in Figure 2, six defected CCNTs are built by periodically introducing the SW and vacancy defects in the different positions of CNT segments. Since the previous studies found that the stress is mainly concentrated at the inner edge of the CCNTs [21,23], the defects are only arranged near the inner edge. According to the relative position of SW defects and the essential heptagon defects in the inner edge of CNT segments, the defects in the middle of two adjacent heptagon defects are identified as the first type SW defects (i.e., (2,3,7,1)/sw1), as shown in Figure 2b. The defects above and under the middle of two adjacent heptagons are identified as the second and third type of SW defects (i.e., (2,3,7,1)/sw2 and (2,3,7,1)/sw3), respectively, as shown in Figure 2c,d. The identification of vacancy defects is similar to that of SW defects, as shown in Figure 2e–g, and the atomic configurations are (2,3,7,1)/v1, (2,3,7,1)/v2, (2,3,7,1)/v3, respectively.

All MD simulations are implemented in the Large-scale Atomic/Molecular Massively Parallel Simulator (LAMMPS) software (version 29Sep2021), which is widely used to investigate the mechanical properties of carbon-based materials [41,42,43]. The Adaptive Intermolecular Reactive Empirical Bond Order (AIREBO) potential field is used to simulate the interatomic forces of CCNTs [44]. In order to avoid the nonphysical results, the initial smaller cutoff radius of 1.7 Å in AIREBO is changed to 2.0 Å [44,45,46]. The stable structures of all CCNTs are obtained through complete relaxation under NPT (constant number of atoms, constant pressure, and constant temperature) ensemble for 500 ps. Also, the temperature of both relaxation and uniaxial tension simulations is set as 1 K to avoid the effect of thermal fluctuation [20]. The periodical boundary condition (PBC) is applied along the z-direction of CCNTs in order to obtain the stable structure of CCNTs without initial stress. Then, all CCNTs are stretched at the constant strain rate of 10^8^/s under NVT (constant number of atoms, constant system volumes, and constant temperature), and the uniaxial tension of CCNTs is carried out by uniformly rescaling the coordinates of all atoms along the z-direction in every 1000 time steps. The time step is set as 1 fs [18,21,22]. The tensile force along the z-axis is calculated based on the virial stress on every atom. The geometrical parameters of the relaxed structure of CCNTs are shown in Table 1, where the effective radius is calculated by [22]:

(1) $$\bar{d}=2\frac{1}{N}\sqrt{\sum_{i=1}^{N}\left({\left(x_{i}-x_{\mathrm{center}}\right)}^{2}+{\left(y_{i}-y_{\mathrm{center}}\right)}^{2}\right)}$$

where *x*_center_ and *y*_center_ are the coordinates of the center in the *x-y* plane of CCNTs containing *N* atoms. *x_i_* and *y_i_* are coordinates of the position of atom *i* in the *x-y* plane.

## 3. Results and Discussion

### 3.1. The Effect of Defects on Elastic Properties of CCNTs

**Figure 3:**
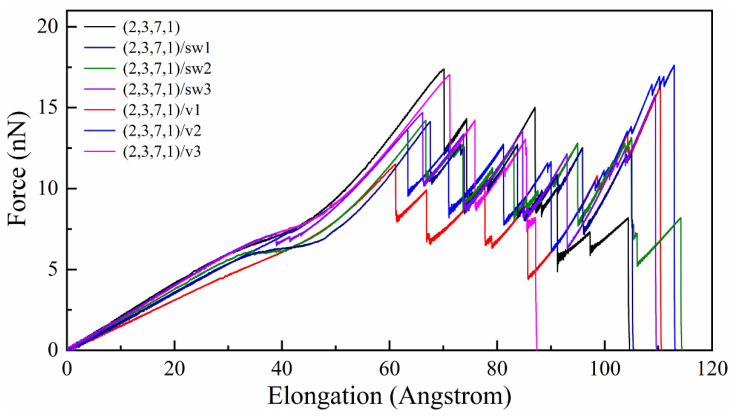
The tensile force–elongation curves of all perfect and defected CCNTs.

**Figure 4:**
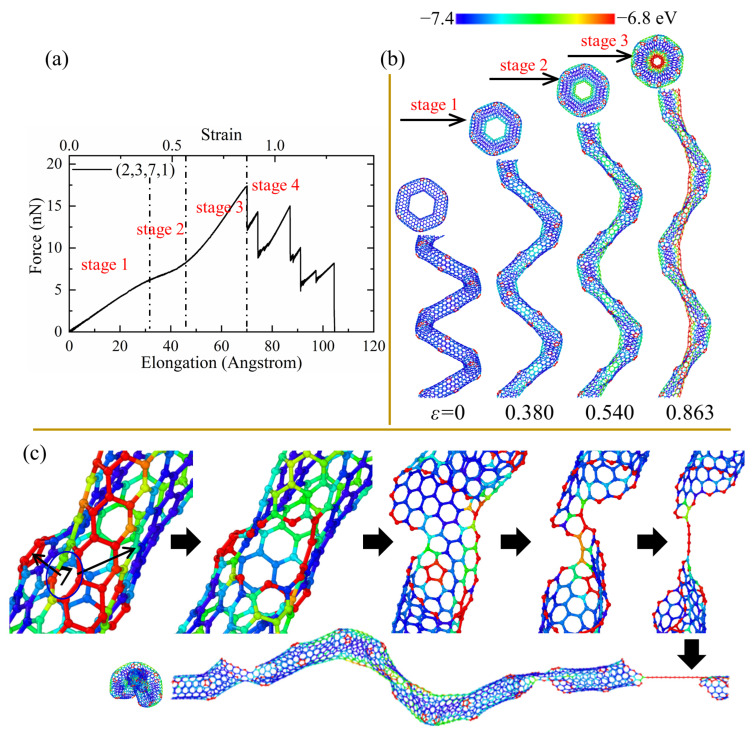
(**a**) The tensile force–elongation curves, (**b**) elastic deformation, and (**c**) fracture process for the perfect CCNT (2,3,7,1). The color of atoms is shown based on the potential.

**Figure 5:**
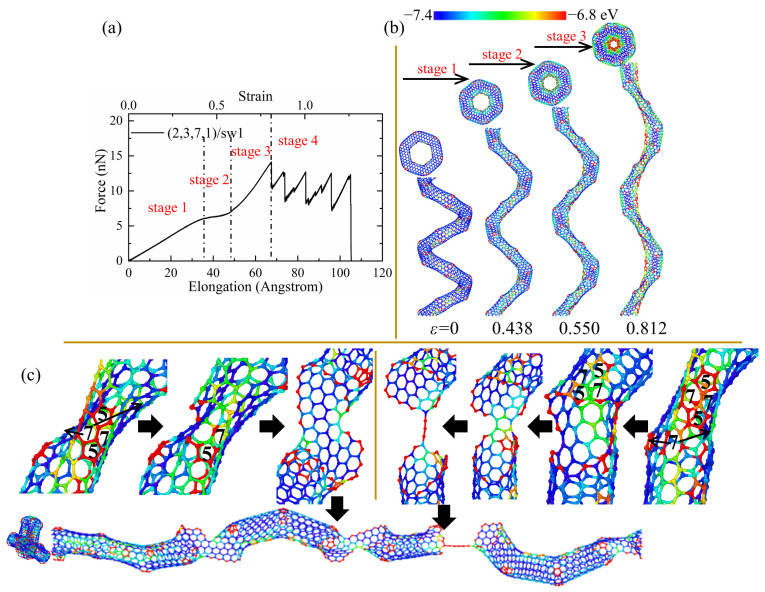
(**a**) The force–elongation curves, (**b**) elastic deformation, and (**c**) fracture process for the CCNT (2,3,7,1)/sw1. The color of atoms is shown based on the potential.

**Figure 6:**
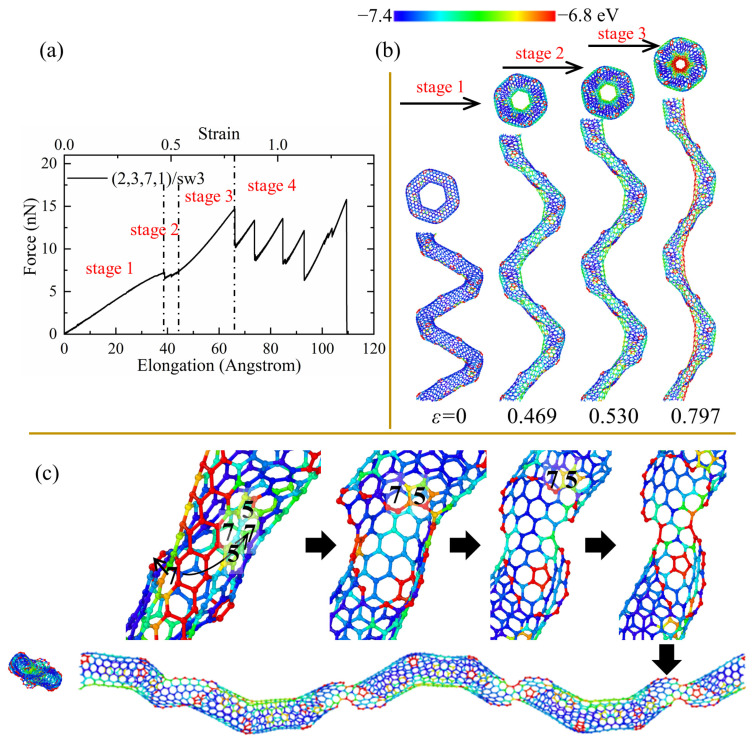
(**a**) The force–elongation curves, (**b**) elastic deformation, and (**c**) fracture process for the CCNT (2,3,7,1)/sw3. The color of atoms is shown based on the potential.

As shown in Figure 3, the tensile force of all CCNTs shows a quasi-linear increase during the elastic range and has three main stages with the increase of the elongation. The first stage is the uniform elongation of CCNTs, which is mainly controlled by the uniform stretching of all C-C bonds. This is indicated by the linear increase of the tensile force in the force–elongation curves. In the second stage, the flattening of CNT segments is observed in Figure 4b, Figure 5b and Figure 6b, which corresponds to the yielding stage in force–elongation curves. In this stage, the rotation of C-C bonds mainly controlled the deformation of CCNTs. In the third stage, the deformation of CCNTs is mainly governed by the stretching of C-C bonds in the inner edge, and the stress is initially concentrated on the inner area, as shown in Figure 4b, Figure 5b and Figure 6b. The linear increase of the force with the increase of strain is also observed in the force–elongation curves. By contrast, the CCNTs with vacancy defects only exhibit uniform stretching deformation, which is indicated by the linear increase of force in force–elongation curves, except for the CCNT (2,3,7,1)/v3 which exhibits a similar deformation pattern with perfect CCNT. Hence, introducing defects can change the elastic deformation pattern of CCNTs, where the SW defects can bring a severe yielding stage while vacancy defects can eliminate the yielding stage.

The elastic limit is defined as the maximum strain where the fracture of bonds appears initially. As shown in Table 2, the introduction of SW or vacancy defects reduces the elastic limit of CCNTs, which can also be seen in Figure 3, where the perfect CCNT has excellent elongation before the failure occurs, indicating that the defects produce the quick emergence of breakage of C-C bonds. Furthermore, the perfect CCNT (2,3,7,1) also possesses the maximum tensile force among all CCNTs during the elastic range, as shown in Figure 3. This is because the stress is uniformly concentrated on the central area of the inner edge of perfect CCNTs, which is indicated by the red nanoribbon in Figure 4b, while introducing the defects leads to the severe stress concentration on the defects, as shown in Figure 5b, which facilitates the breakage of C-C bonds. CCNTs with vacancy defects also show a similar phenomenon, except for the CCNTs with the third type of vacancy defects. Among CCNTs with SW defects, (2,3,7,1)/sw1 possesses the maximum elastic limit while the (2,3,7,1)/sw3 has the minimum elastic limit where the defects are distributed below the middle of CNT segments, as shown in Figure 6b. The opposite pattern is observed for CCNTs with vacancy defects where the (2,3,7,1)/v3 has the maximum elastic limit, which explains that the vacancy defects under the CNT segments cannot obviously affect the deformation of CCNTs.

During the elastic stage, the spring constant can be determined by fitting the linear force-displacement curves within the strain range of 0% to 2%. It can be seen from Table 2 that for the CCNTs with SW defects, CCNTs (2,3,7,1)/sw1 with the defects at the center of CNT segments possesses the minimum spring constant while the maximum spring constant is observed for (2,3,7,1)/sw3 with defects underlying CNT segments. The same pattern is also observed for CCNTs with vacancy defects. This is because the defects under the CNT segments cannot distinctly affect the deformation pattern of CCNTs since the stress concentration occurs on the middle area of the inner edge.

In order to study the ability of energy storage in the elastic range, the elastic gravimetric energy density *E*/*m* (J/g) (reversible energy stored per unit mass) is calculated. *E* is obtained by calculating the area under the force–elongation curves, and *m* is the total mass of CCNTs. As shown in Table 2, the defected CCNTs possess lower energy storage ability than perfect CCNTs. This is because introducing defects in CCNTs reduces the force to stimulate crack initiation and elastic limit simultaneously, as shown in Figure 3. Furthermore, among defective CCNTs, their energy storage capacity also shows a difference due to the different positions of defects. (2,3,7,1)/sw3 can store most energy (1177.96 J/g) during the elastic range because the SW defects are arranged under the CNT segments, which cannot obviously reduce the force of crack initiation of CCNTs, while the (2,3,7,1)/sw1 has the relatively poor energy storage capacity (1089.50 J/g) since the introducing defects on the central area cause the high-stress concentration on the defects, inducing the fast breakage of CNT segments. The same pattern is also observed for the CCNTs with vacancy defects.

### 3.2. The Effect of Defects on the Plasticity and Fracture Processes of CCNTs

In order to investigate the effect of SW and vacancy defects on the plasticity and fracture pattern of CCNTs, the maximum strain, irreversible gravimetric energy density, and fracture process are calculated and examined as follows.

As shown in Figure 3, the tensile force shows strong fluctuation and a decreasing trend in the force–elongation curves after the initial cracks. Such fluctuation is induced by the partial fracture of joints linking the CNT segments, as shown in Figure 4c. However, the tensile force and elongation show different patterns for perfect and defected CCNTs. The perfect CCNT (2,3,7,1) possesses relatively poor ductility, as shown in Figure 3, because of the uniform concentration of the stress on the inner edge indicated by the red nanoribbon in Figure 4b. As can be seen from Figure 4a, the oscillation and obvious decrease trend are observed for the force in the force–elongation curves after the initiation of cracks. Such oscillation is caused by the partial fracture of CNT segments, as shown in Figure 4c, where cracks initiate from the heptagon at the joints, and breakages of the joints repeat three times to generate a long single carbon atom chain before complete rupture. The poor ductility and the loads bearing capacity after initiation of fracture cause the poor energy absorbing capacity of perfect CCNTs (929.88 J/g), as shown in Table 2.

As can be seen from Table 2 and Figure 3, the defective CCNTs with the second and third types of SW defects exhibit better ductility than the perfect CCNTs, while the CCNTs with the first and second types of vacancy defects possess better ductility. For the (2,3,7,1)/sw1, the high concentration of stress on the SW defects is observed, as shown in Figure 5b, where there is no red nanoribbon formed like perfect CCNT, which induces the premature breakage of C-C bonds. Hence, the ductility of the (2,3,7,1)/sw1 is poorer than that of (2,3,7,1). However, there is no obvious decrease trend for the force after the fracture initiation, as shown in Figure 5a, which leads to the great energy absorbing capacity (over 1082 J/g) during the fracture process. From Figure 5c, it can be seen that the deformation pattern also shows a difference where cracks initiate from the heptagon of SW defects and propagate towards the pentagon of SW defects. After the crack cuts through the CNT tubes, a carbon nano-band is formed, and other pair of heptagon and pentagon of SW defects remains unbroken. Before the complete rupture, a different partial fracture pattern is observed, as shown in Figure 5c, where the crack initially propagates from the heptagon rings at the CNT segment joints, and the SW defects remain unchanged to form a short single carbon chain. For the CCNT (2,3,7,1)/sw3, there is a different deformation pattern because the SW defects are distributed under the CNT segments, which cannot affect the stress concentration, obviously. The stress is still uniformly concentrated at the inner edge, which is demonstrated by the red nanoribbon in Figure 6c. The crack initiates from the heptagon at the joints and propagates towards the SW defects, cutting through one pair of heptagon–pentagon defects. Unlike (2,3,7,1)/sw1, there is no single carbon chain formed. Remarkably, after the initiation of the crack, the force does not decrease distinctly, and the force before complete rupture is even larger than the initial failure point, as shown in Figure 6a, which can help to absorb more energy during the fracture process (1308 J/g).

**Figure 7:**
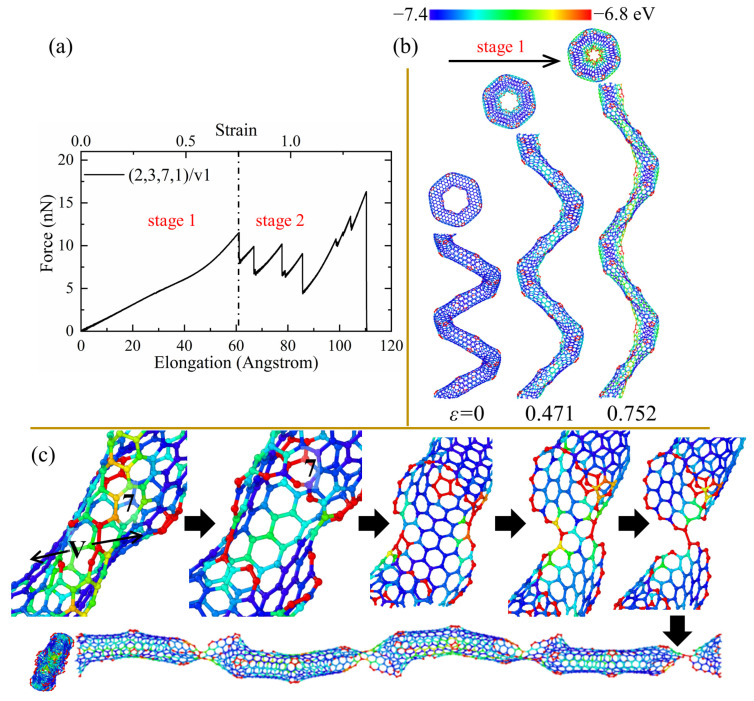
(**a**) The force–elongation curves, (**b**) elastic deformation, and (**c**) fracture process for the CCNT (2,3,7,1)/v1. The color of atoms is shown based on the potential.

**Figure 8:**
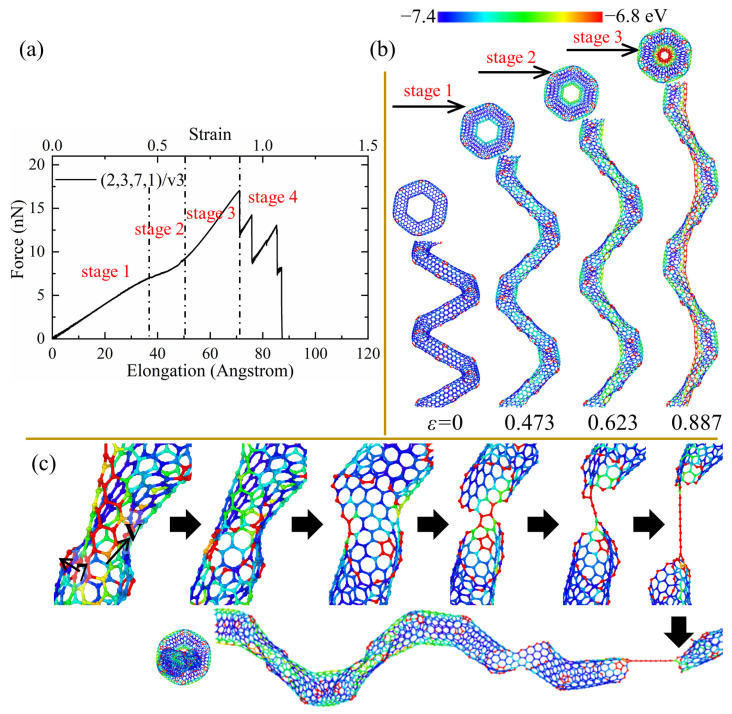
(**a**) The force–elongation curves, (**b**) elastic deformation, and (**c**) fracture process for the CCNT (2,3,7,1)/v3. The color of atoms is shown based on the potential.

As for (2,3,7,1)/v1, it can be seen that a high concentration of stress appears on the carbon atoms near vacancy positions in the central area of the inner edge in Figure 7b. The partial fracture always starts from the vacancy defects and cuts through the CNT segments from the central position while maintaining the heptagons at the CNT segment joints, as shown in Figure 7c. Hence, the CNT segments can unfold efficiently to form a straight structure where the broken CNT segments are only linked by two hexagonal rings instead of the nanoribbons in the (2,3,7,1) and (2,3,7,1)/sw3. The efficient unfolding of CNT segments leads to better ductility (over 130%). Such sequential fractures of the CNT segments also lead to the oscillation of the force in the force–elongation curves and can consume more energy through the fracture of more bonds, which is indicated by the irreversible energy density (1249.20 J/g) in Table 2. It is also worth noting that the force to completely break the whole structure of (2,3,7,1)/v1 is much larger than the force of the initial breakage, as shown in Figure 7a, which means that (2,3,7,1)/v1 can still work to bear a large load before complete rupture. A similar deformation pattern is also observed for the (2,3,7,1)/v2 while (2,3,7,1)/v3 shows a different pattern. As shown in Figure 8b, the introduction of the third type of vacancy defects does not affect the uniform stress concentration, which is indicated by a red nanoribbon on the inner edge of (2,3,7,1)/v3. Therefore, like the perfect CCNT (2,3,7,1), the fracture initiates from the heptagon rings at the CNT joints and propagates to the pentagon rings at joints and vacancy defects. Since the stress is distributed uniformly along the inner edge, only two joints between CNT segments unfold, and the top view of the broken (2,3,7,1)/v3 remains the hexagonal shape. The insufficient breakages of the joints lead to poor ductility (108.58%) and cause the weaker fluctuation of force in the tension force–elongation curves, as shown in Figure 8a. Therefore, the energy absorbing capacity (501.37 J/g) of (2,3,7,1)/v3 is much poorer than other CCNTs, which can be seen in Table 2.

## 4. Conclusions

The effects of the Stone–Wales and vacancy defects on the tensile mechanical performance of CCNTs are studied through MD simulations in this research. Based on the perfect CCNT constructed by CNT segments with a charity of (6, 6), six defective CCNTs with various positions in the CNT segments are constructed. The mechanical properties and deformation mechanism show obvious differences between perfect and defective CCNTs with various defect positions. The conclusion can be summarized as follows:

(a) The defected CCNTs generally possess slightly smaller stiffness (from 1.48 nN/nm to 1.93 nN/nm) and smaller elastic limit (75.2% to 88.7%) than perfect CCNT (1.89 nN/nm and 86.3%, respectively) because introducing the defects changes the stress distribution and causes premature breakages of C-C bonds. Also, the energy storage capacity of defected CCNTs (842.74 J/g to 1433 J/g) is generally lower than that of perfect CCNT (1412.59 J/g) due to the smaller elastic limit and force of crack initiation.
(b) The ductility of defected CCNTs is generally better than that of perfect CCNTs, and more energy is consumed during the fracture progression for the defective CCNTs due to the high residual load-carrying capacity and large plastic deformation. Among the defected CCNTs, both (2,3,7,1)/sw3 and (2,3,7,1)/v2 possess the better stiffness (1.93 nN/nm and 1.63 nN/nm, respectively), elastic limit (79.7% and 77.9%, respectively), ductility (up to 138.9%) and excellent energy absorbing capacity (up to 1539.93 J/g).
(c) The deformation pattern of defected CCNTs is obviously different under tensile loads. There is an obvious yielding stage within the elastic range in the tensile force–elongation curves of CCNTs with the SW defects, which is not observed for CCNTs with vacancy defects. During the fracture process, the partial fracture usually initiates from the defect position, which can break the CNT segments sufficiently for (2,3,7,1)/sw1 and (2,3,7,1)/v1 with the defects at the center of the CNT segments. The deformation process of (2,3,7,1)/v3 is similar to that of perfect CCNT since the defects under the CNT segments do not affect the stress concentration on the central area of the inner edge of CCNTs.

The findings in this research provide an understanding of the controllable mechanical performance and underlying failure mechanism of CCNTs with SW and vacancy defects through adjusting the defect position, which is valuable for manufacturing CCNTs with superior properties according to different applications.

## Figures and Tables

**Table 1:** Geometrical parameters of CCNTs.

CCNTs	Number of Atoms	Tube Radius (Å)	Effective Radius (Å)	Pitch Length (Å)
(2,3,7,1)	1800	4.10	13.12	40.65
(2,3,7,1)/sw1	1800	4.10	13.15	41.23
(2,3,7,1)/sw2	1800	4.10	13.18	41.56
(2,3,7,1)/sw3	1800	4.10	13.17	41.54
(2,3,7,1)/v1	1788	4.10	13.17	40.64
(2,3,7,1)/v2	1788	4.10	13.15	40.66
(2,3,7,1)/v3	1788	4.10	13.16	40.10

**Table 2:** Tensile mechanical properties of CCNTs.

CCNTs	Spring Constant (nN/nm)	Elastic Limit	Elastic Energy Density (J/g)	Irreversible Energy Density (J/g)	Maximum Strain
(2,3,7,1)	1.89	0.86	1412.59	929.88	1.285
(2,3,7,1)/sw1	1.70	0.81	1089.50	1082.56	1.264
(2,3,7,1)/sw2	1.75	0.81	1103.89	1310.32	1.381
(2,3,7,1)/sw3	1.93	0.80	1177.97	1308.24	1.318
(2,3,7,1)/v1	1.48	0.75	842.74	1249.20	1.358
(2,3,7,1)/v2	1.63	0.78	1042.32	1539.93	1.389
(2,3,7,1)/v3	1.82	0.887	1433.42	501.37	1.085

## Data Availability

The data presented in this study are available on request from the corresponding author.
